# Low observed perioperative complication rates associated with surgical sterilization of 1,011 heartworm-infected dogs using standard, unmodified anesthesia and surgical protocols

**DOI:** 10.1186/s13071-026-07514-x

**Published:** 2026-08-27

**Authors:** Jessie L. Dyer, Linda S. Jacobson, Maria A. Serrano, Brian A. DiGangi, Joey Friedman, Hannah M. Garcia, Mia K. Cuccinello, Julie K. Levy

**Affiliations:** 1 Shelter Medicine Program, University of Florida College of Veterinary Medicine, Gainesville, FL USA; 2 Toronto Humane Society, Toronto, ON Canada; 3 Miami-Dade Animal Services, Miami, FL USA; 4 EveryPet, Jacksonville, FL USA

**Keywords:** Canine, Heartworm, *Dirofilaria immitis*, Perioperative complications, Surgical sterilization, Spay–neuter

## Abstract

**Background:**

Shelters routinely sterilize dogs before adoption using standardized, high-volume protocols. Many also admit heartworm-positive dogs, which often experience reduced adoption appeal, longer shelter stays and higher euthanasia risk. Sterilizing these dogs before melarsomine treatment can expedite placement, but safety data on using standard, unmodified protocols remain limited. This study investigated perioperative complications in dogs with mild to moderate heartworm disease (HWD) undergoing sterilization prior to melarsomine therapy.

**Methods:**

This was a retrospective observational study of 1,011 clinically stable shelter dogs with Class 1–2 HWD treated between 2014 and 2024. All received a standardized anesthetic protocol of dexmedetomidine, ketamine, butorphanol, and isoflurane. Surgeries were classified as routine or complex. Complications were categorized as major (life-threatening events, severe surgical problems, or aborted procedures) or minor (self-limiting issues or those requiring minimal, non-surgical intervention).

**Results:**

The study included 1,011 dogs (440 females, 571 males); 92.2% underwent routine spay-neuter procedures. No perioperative deaths occurred. Twelve dogs (1.2%) experienced major complications, including three anesthetic events of unclear etiology that could not be definitively attributed to HWD, and nine surgical-site complications requiring revision. Minor complications occurred in 39 dogs (3.9%), primarily superficial incisional issues (3.6%) and transient hematuria (< 1%). Most complications were superficial surgical‑site issues commonly observed after routine sterilization, and there was no evidence of an unexpectedly high rate of cardiopulmonary events in this heartworm‑positive cohort.

**Conclusions:**

Unmodified high-volume anesthetic and surgical protocols were associated with low perioperative complication rates in a selected population of dogs with Class 1–2 HWD. Sterilizing dogs with mild to moderate HWD before adulticide therapy appears safe when using standard spay–neuter protocols and supports earlier adoption, improved shelter throughput, and efficient resource use without compromising patient safety.

**Graphical Abstract:**

**Figure:**
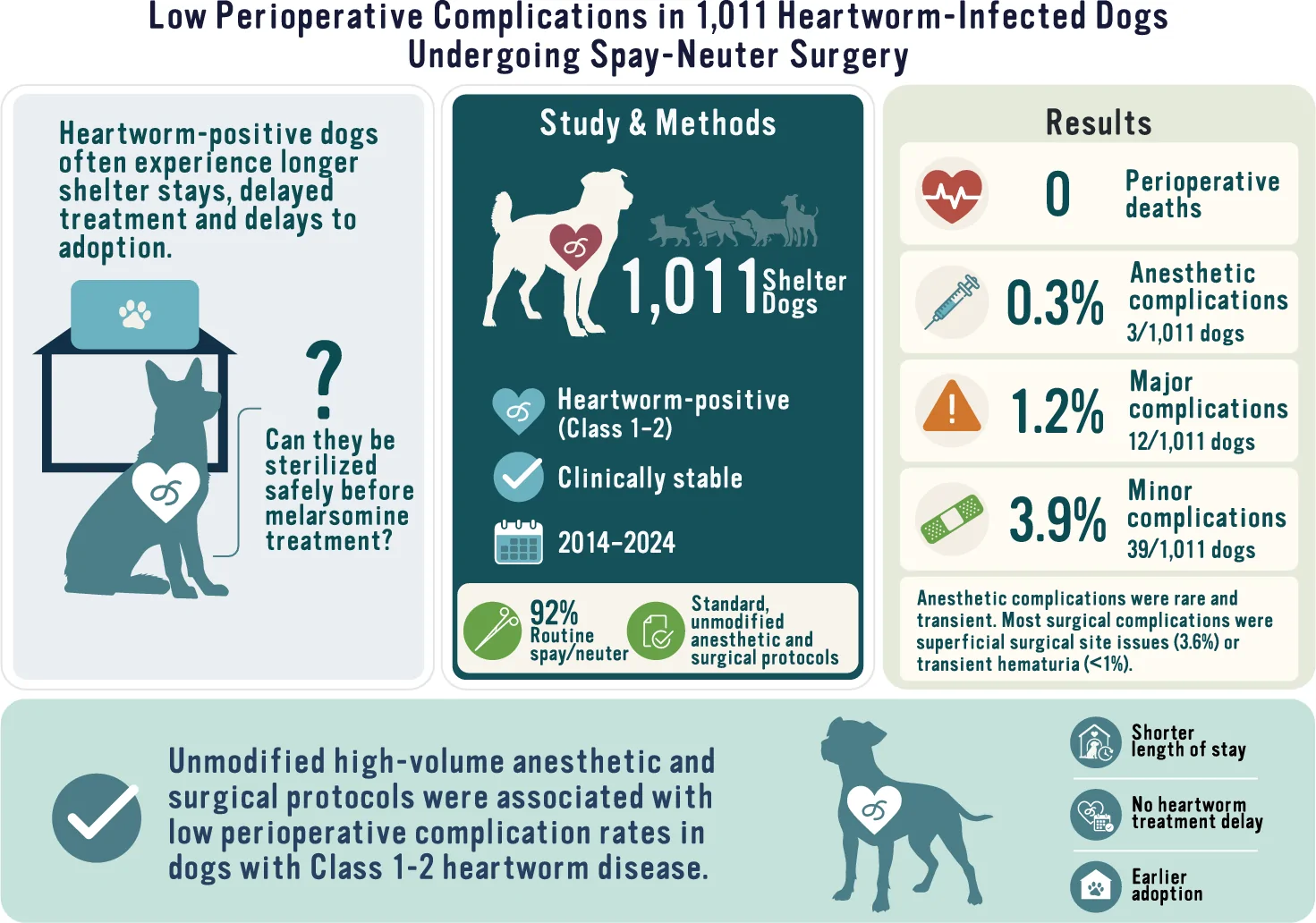

## Background

Surgical sterilization of companion cats and dogs is routine in veterinary medicine. In 2008, the Association of Shelter Veterinarians published spay–neuter guidelines described as “applicable and achievable” across all veterinary practice environments [1]. The guidelines provided recommendations for preoperative assessment, anesthetic management, surgical procedures, and postoperative care using current principles of anesthesiology, surgical practice, critical care medicine, and infection control. The 2016 update defined high-quality, high-volume spay–neuter (HQHVSN) services as “efficient surgical initiatives that meet or exceed veterinary medical standards of care in providing accessible, targeted sterilization of large numbers of cats and dogs to reduce their overpopulation and subsequent euthanasia”[2].

While these guidelines establish a clinical standard, their application in animal shelter environments is shaped by unique operational constraints. Animal shelters strive to allocate resources to help the greatest number of animals in the shortest amount of time while safeguarding individual animal welfare [3, 4]. Shelter dogs often arise from resource-scarce conditions or lack previous preventive healthcare, leaving them at risk for the higher rates of HWD than typically observed at private veterinary practices. In addition, shelter dogs are more likely to be intact than owned dogs [5].

Beyond the routine demands of surgical sterilization, shelters in heartworm-endemic regions face an additional and significant clinical challenge. Canine heartworm disease (HWD) adds cost and time required for diagnosis, treatment, and follow-up [4, 6, 7]. Heartworm (HW) prevalence is higher in unowned dogs and those admitted to animal shelters compared with owned dogs [8–10]. Adopters may be reluctant to take on untreated HW-positive dogs due to the cost of treatment, potential for complications, and prolonged period of required exercise restriction. In shelters, insufficient resources, limited access to veterinary care, lack of space, and reduced adopter appeal keep HW-positive dogs at higher risk of euthanasia [4]. Surgical sterilization prior to melarsomine treatment promotes early adoption and supports shelter operational priorities, but little is known about the safety of using unmodified high-volume protocols in HW-positive dogs.

These epidemiologic realities raise important questions about the safety of anesthesia and surgery in HW-positive dogs. Adult HWs are found primarily in the pulmonary arteries, and infection results in endarteritis, fibrosis, and increased vascular resistance [7, 11–13]. Higher worm burdens and chronic infections can lead to pulmonary hypertension, pulmonary thromboembolism, and pneumonitis, and may progress to right-sided heart failure [7, 14]. These pathophysiologic changes directly influence anesthetic risk through altered pulmonary vascular resistance and impaired gas exchange. Cardiac and pulmonary function are critical considerations when assessing anesthetic risk, leading to concerns about performing elective procedures on HW-positive dogs.

Reduced cardiac output from cardiac-depressant anesthetic drugs, combined with reduced functional lung volume, pulmonary hypertension, or arrhythmias, could result in hypoxia and possibly increase the risk of pulmonary thromboembolism [15, 16]. HW-positive dogs more frequently exhibit laboratory evidence of prothrombotic, fibrinolytic, and thrombocytopenic tendencies compared to uninfected dogs, adding to concerns about the safety of surgical procedures [17–20]. Co-infection with thrombocytopenia-inducing vector-borne pathogens represents an additional potential risk factor [21–23]. In a survey of shelter veterinarians’ HW management practices, 82.1% of 158 respondents stated that they performed elective surgical sterilization prior to adulticide treatment (“after diagnosis, prior to treatment” 44.3%; “after beginning doxycycline” 23.4%; “prior to diagnosis” 8.2%; “after completing doxycycline” 6.3%). Only 8.9% of respondents typically performed sterilization procedures after melarsomine administration [6].

Despite these theoretical concerns, limited empirical data suggest that selected HW-positive dogs may tolerate anesthesia and surgery without complication. Two studies have evaluated the safety of pre-melarsomine sterilization in HW-positive dogs with mild or absent clinical signs [15, 17]. The first report assessed 15 hemodynamically stable HW-positive shelter dogs with no or mild clinical signs of HWD. Presurgical evaluation included HW testing, complete blood count, serum chemistry, electrocardiogram, and thoracic radiographs. A cardiovascular-sparing anesthetic protocol was used, consisting of premedication with acepromazine, butorphanol, and a nonsteroidal anti-inflammatory; tiletamine–zolazepam or ketamine–diazepam induction; and maintenance with isoflurane or sevoflurane. No perioperative complications were observed [15]. In the second report, 20 asymptomatic HW-positive dogs and 20 age- and weight-matched HW-negative dogs underwent elective sterilization at a HQHVSN clinic. The study assessed perioperative coagulation tendencies, intraoperative bleeding, and surgical times. Anesthesia was induced with the clinic’s standardized protocol of ketamine–diazepam followed by buprenorphine and maintenance with isoflurane. Although HW-positive dogs showed laboratory evidence of hypercoagulability, no significant differences were identified in intraoperative blood loss or surgical time [17]. These studies were limited by small sample sizes (*n* = 15–40) and preselection of clinically stable patients.

Taken together, existing studies, anecdotal experience, and expert opinion support the practice of pre-adulticide sterilization in dogs with mild to moderate HWD [15–17, 24]; however, robust data from large cohorts using standard, unmodified protocols are lacking. Such cases would generally be categorized in the veterinary adaptation of the American Society of Anesthesiologists (ASA) Physical Status Classification System as ASA II (slight risk): “A patient with mild systemic disease or a minor systemic disturbance. The animal is able to compensate for the condition.” To our knowledge, however, no studies have evaluated pre-adulticide surgical sterilization of a large cohort of HW-positive dogs using standard anesthetic and surgical protocols. The objective of this retrospective study was to assess mortality and perioperative complications, particularly those attributable to HWD, in a large cohort of dogs with Class 1 or 2 HWD undergoing routine sterilization in a high-volume setting.

## Methods

### Facility

This retrospective study examined surgical sterilization data from HW-positive dogs admitted to Miami-Dade Animal Services from February 1, 2014, to July 3, 2024. Final shelter outcomes were determined in September 2025. Miami-Dade Animal Services is a municipal shelter serving Miami-Dade County, which covers 1,946 square miles of land mass and serves a population of 2.7 million residents [25]. The county is located along the south–easternmost coast of Florida in a tropical monsoon climate, where *Dirofilaria immitis* is endemic. During the study period, approximately 8,000–10,000 dogs were admitted to the shelter annually.

### Study population

Within this shelter setting, electronic medical records were reviewed to identify dogs meeting predefined inclusion criteria. Records housed in the shelter’s Chameleon® Shelter Case Management System were searched to identify dogs that were sterilized prior to melarsomine treatment. Dogs were included if they were at least 7 months of age, tested positive for HW antigen (Ag) at intake, had clinical signs consistent with American Heartworm Society guidelines Class 1 (asymptomatic or cough) or Class 2 HWD (cough, activity intolerance, abnormal lung sounds), and were surgically sterilized within 180 days of HW diagnosis. Severity of HWD was based on physical examination alone; additional laboratory evaluation, radiography, and echocardiography were not performed. Dogs were excluded if they had clinical signs of severe HWD, including Class 3 (signs of right heart failure) or Class 4 (caval syndrome). However, reliance on clinical signs alone may have allowed the condition of some more severely affected dogs to remain undetected.

### Canine shelter medicine protocols

#### Preventive healthcare

All dogs received a physical examination by a veterinary technician upon shelter admission, during which they were treated for external and gastrointestinal parasites and received age-appropriate vaccines against distemper virus, adenovirus, parvovirus, parainfluenza, *Bordetella bronchiseptica*, and rabies. Veterinary staff scanned all dogs for identification microchips and implanted one if not already present. A veterinary examination was scheduled for dogs with clinical concerns not covered by protocols delegated to veterinary technicians. Sexually intact dogs were scheduled for the next available surgery time if considered to be stable candidates for sterilization.

#### Heartworm testing, preventive, and treatment

Dogs were screened for HWs using a point-of-care Ag test (IDEXX SNAP® Heartworm Test or IDEXX SNAP® 4Dx® Test, IDEXX Laboratories, Westbrook, ME, USA). Dogs with positive test results were also tested for microfilariae (Mf) using direct microscopy. Dogs were considered HW-positive based on Ag test results, regardless of the Mf results. All dogs received a macrocyclic lactone HW preventive. Monthly ivermectin or milbemycin was administered from 2014 to March 2023 and sustained-release injectable moxidectin thereafter. Dogs with positive Mf tests were treated with dexamethasone (0.15 mg/kg SC) and diphenhydramine (2 mg/kg SC) prior to macrocyclic lactone administration. Doxycycline (10 mg/kg PO), or minocycline during periods of unavailability, was administered once daily for 28 days to HW-positive dogs. HW-positive dogs were scheduled for a 2-dose or 3-dose melarsomine adulticide treatment protocol after sterilization surgery.

#### Anesthesia and surgical sterilization

Dogs received a standardized shelter anesthetic protocol applied uniformly regardless of heartworm status. Preanesthetic examinations were performed by a veterinarian on the day of surgery prior to induction. Dogs received a combination of dexmedetomidine (10 µg/kg), ketamine (2 mg/kg), and butorphanol (0.2 mg/kg) (DKT). From 2014 to 2018, half of this mixture was administered intramuscularly (IM) and half intravenously (IV); from 2019 onward, the total dose was administered IM. Dogs were then intubated and maintained on isoflurane and oxygen. All dogs received preoperative carprofen (4.4 mg/kg SC), and postoperative opioids were administered at the attending veterinarian’s discretion. Intravenous fluid therapy (10–20 ml/kg/h) was administered intraoperatively to pregnant dogs and those with conditions such as pyometra, at the discretion of the surgeon. Surgical procedures were performed using accepted HQHVSN techniques [2].

### Categorization of surgical procedures and perioperative complications

Complication classification was performed retrospectively and may be subject to misclassification bias. Surgical procedures were categorized as follows:

- Routine: Sterilization procedures in nongravid dogs with normal and healthy reproductive anatomy
- Complex: Sterilization procedures for pregnancy, pyometra/hydrometra, cryptorchidism, scrotal ablation, or additional surgical procedures performed concurrently

A matrix for defining complications based on timing, type, and severity was developed prior to medical records review as follows:

- Timing: Preoperative, intraoperative, postoperative
- Severity (major): Death, cardiac arrest, apnea, surgery aborted, severe hemorrhage, delayed anesthetic recovery, surgical revision required, other life-threatening conditions
- Severity (minor): Non-life-threatening conditions that resolved without medical intervention or with short-term, non-surgical intervention
- Type: Anesthetic, hemorrhage, surgical site

### Statistical analysis

Descriptive statistics for dog age, weight, sex, type of surgery, complications, and final outcomes were calculated for categorical data by counts and percentages, and for continuous data by medians and ranges. Surgical-site complication rates in males vs. females were compared using the chi-square test (comparisons with frequency ≥ 5 in all contingency table cells) or the Fisher’s exact test (tables with < 5 results in any cell) as appropriate. No adjustments for multiple comparisons were made. Statistical significance was set at *P* < 0.05.

## Results

### Animals and surgical procedures

A total of 1,011 HW-positive dogs met the study inclusion criteria. Of those, 974 were tested for Mf, with 425 (43.6%) testing positive and 549 (56.4%) testing negativs. The cohort included 440 females (43.5%) and 571 males (56.5%). The median age at HW diagnosis was 36 months (range 7–144 months), and the median intake weight was 23.8 kg (range 2.3–72.6 kg) (Table 1). In most dogs (982/1,011; 97.1%) doxycycline (10 mg/kg PO q 24 h) was initiated prior to surgery (median 8 days; range 1–174 days; IQR 28). Twenty-nine (2.9%) received no doxycycline before surgery, 470 (46.5%) received 1–7 days, 219 (21.7%) received 8–27 days and 293 (29.0%) completed the full course before surgery. Twelve dogs received their first melarsomine injection uneventfully during postoperative anesthetic recovery.

**Table 1:** Characteristics of 1,011 heartworm-positive dogs undergoing sterilization at an animal shelter using standard, unmodified anesthesia and surgical protocols

Variable	Category	*n* (%)
Age (*n* = 1,011)	7–23 months	201 (19.9%)
	24–35 months	252 (24.9%)
	36–47 months	266 (26.3%)
	> 48 months	283 (28.0%)
	Mature, age not estimated	9 (< 1%)
Weight (*n* = 1,011)	0–10.0 kg	110 (10.9%)
	10.1–25.0 kg	475 (47.0%)
	25.1–45.0 kg	412 (40.8%)
	> 45.0 kg	14 (1.4%)
Spay procedure (*n* = 440)	Routine spay	393 (89.3%)
	Pregnancy	22 (5.0%)
	Pyometra or hydrometra	11 (2.5%)
	Spay with additional procedure	14 (3.2%)
Castration Procedure (*n* = 571)	Routine castration	539 (94.4%)
	Cryptorchid castration	21 (3.7%)
	Scrotal ablation	2 (0.4%)
	Castration with additional procedure	9 (1.6%)

Routine spay procedures were performed in 393 of 440 female dogs (89.3%) (Table 1). The remaining procedures in females were categorized as complex due to pregnancy (22; 5.0%), pyometra or hydrometra (11; 2.5%), or concurrent procedures at the time of spay (14; 3.2%). Routine castrations were performed in 539 of 571 male dogs (94.4%). The remaining procedures were categorized as complex due to cryptorchidism (21; 3.7%), scrotal ablation (2; 0.4%), or castration performed concurrently with additional surgical procedures (9; 1.6%).

### Perioperative complications

Perioperative outcomes were assessed for all included procedures. No perioperative mortalities occurred. Perioperative complications were reported in 51 dogs (5.0%). All except two occurred postoperatively, and most involved surgical-site complications (45/51; 88%) (Table 2). The overall complication rate was significantly higher in males (42/571; 7.4%) than in females (9/440; 2.0%) (*χ*2 = 14.62; *df* = 1; *P* = 0.0001). This difference was driven primarily by surgical factors, with a higher frequency of postoperative surgical-site complications following castration (41, 7.2%) compared with spay (4, 0.9%) (*P* < 0.0001; OR = 10.6; 95% CI = 7.7–14.7). All dogs recovered fully. Due to the low number of complications potentially associated with HWD (3 cases), complication rates were not adjusted for potential confounders, such as procedure type or bodyweight.

**Table 2:** Perioperative complications in 1,011 heartworm-positive dogs undergoing sterilization surgery at an animal shelter using standard, unmodified anesthesia and surgical protocols

Category	*n* (%)
Total complications	51 (5.0%)
Perioperative mortality	0 (0%)
Timing of complication	
Preoperative	1 (< 1%)
Intraoperative	1 (< 1%)
Postoperative	49 (4.8%)
Complication severity and type	
Major	
Anesthetic	3 (< 1%)
Surgical site—revision required	9 (< 1%)
Minor	
Postoperative hematuria	3 (< 1%)
Surgical site—managed medically	36 (3.6%)
Concurrent surgery performed	
Hernia repair^a^	7 (< 1%)
Lumpectomy	9 (< 1%)
Mastectomy	2 (< 1%)
Other procedures^b^	5 (< 1%)

^a^Includes one patient with hernia repair plus cystostomy

^b^Other concurrent procedures included aural hematoma repair, cystotomy, femoral head ostectomy, tail amputation, toe amputation, and wound repair

#### Major complications

Major complications occurred in 12 dogs (1.2%) (Table 2). These included three anesthetic complications that might have been linked to HWD but also could have been unrelated, given the varied presentations and low overall incidence of anesthetic problems. One dog exhibited watery discharge from the nose and mouth during surgery, accompanied by a transient abnormal respiratory pattern. Another dog experienced delayed anesthetic recovery accompanied by mild hypothermia, and the third dog developed apnea at induction. Anesthesia was terminated in the apneic dog, which subsequently underwent uneventful spay surgery 6 weeks later. Surgeries were completed on the other two dogs with anesthetic complications. Other major complications included incision site issues requiring surgical revision in nine dogs (< 1%), seven of which included postoperative scrotal hematomas that were resolved by subsequent scrotal ablation (Table 2). No unusual intraoperative or postoperative hemorrhage was reported.

#### Minor complications

Minor complications occurred in 39 dogs (3.9%), including superficial incision site issues that resolved without surgical intervention in 36 (3.6%) and transient postoperative hematuria in three (< 1%) dogs (Table 2).

### Final shelter outcomes

Most dogs were adopted (946/1,011, 93.6%). Additional live outcomes included return to owner (32; 3.2%) and transfer to another organization (17; 1.7%). Eight dogs (< 1%) were euthanized for reasons unrelated to HWD, the surgical procedures, or complications. Two dogs escaped, and no final outcome was recorded in the shelter software system for one dog. Five dogs (< 1%) remained in shelter care as of October 2025.

## Discussion

In this large cohort of 1,011 dogs with Class 1–2 (mild to moderate) HWD, perioperative outcomes following surgical sterilization were favorable. Both routine and complex sterilization procedures performed without modification of standard HQHVSN anesthesia and surgical protocols in this cohort of clinically stable dogs were associated with low complication rates. There was no mortality, and cardiopulmonary complications, the primary concern regarding anesthesia and surgery in HW-positive dogs, were observed in 0.3% of dogs.

Only three of the 1,011 dogs experienced anesthetic complications, and all were managed successfully without intensive interventions or lasting sequelae. Although rare, anesthetic complications may be under-detected due to the retrospective design and limited perioperative monitoring data recorded in the procedure templates. Most perioperative complications were superficial surgical‑site issues commonly observed after routine sterilization, and there was no evidence of an unexpectedly high rate of cardiopulmonary events in this heartworm‑positive cohort.

An additional consideration in the perioperative management of HW-positive dogs is the timing of adulticide therapy relative to anesthesia. Twelve dogs received melarsomine during anesthetic recovery. Although the sample was small, no complications were observed. This approach avoids the need for separate sedation and analgesia, reduces patient stress, and decreases the use of shelter resources. No complications were observed in this small subset, suggesting this may be a feasible strategy that warrants further study.

This study demonstrates that in dogs with clinically asymptomatic to moderate HWD, pre-adulticide anesthesia and surgery can proceed using routine anesthetic protocols and without routine additional diagnostics in this particular setting. These findings are reassuring, as HW status is unknown for many dogs admitted for sterilization surgery. In one HQHVSN clinic in an endemic region of the southern United States, 11.7% of dogs tested HW-positive at the time of their sterilization appointment [23]. Thus, veterinarians working in HW-endemic regions may routinely and unknowingly encounter dogs with HWD, particularly if practicing in shelter settings, where medical history of dogs is unknown, or in low-cost clinics serving clients with limited access to veterinary care. Further study is warranted to determine if additional screening may benefit higher risk populations.

The near-universal administration of doxycycline and macrocyclic lactones prior to surgery may have mitigated perioperative risk and represents a key confounding variable. These adjunctive treatments, particularly doxycycline, reduce inflammation and the severity of thromboembolic events following worm death, primarily through depletion of *Wolbachia*, a pro-inflammatory bacterial endosymbiont of *D. immitis* that is released from dead and dying worms [26–28]. It is unknown whether, or how rapidly, administration of macrocyclic lactones and doxycycline may protect against anesthetic complications in HW-positive dogs. However, anesthesia is not expected to result in worm death, and the benefits of doxycycline are primarily related to the effects of dead and dying worms [26]. The median time from initiation of doxycycline to surgery was 8 days, while the recommended duration of doxycycline in HW treatment is 28 days [7]. We are not aware of quantitative studies of depletion of *Wolbachia* in heartworms or resolution of related pulmonary inflammation earlier than a month after initiation of doxycycline. Initiating adjunctive treatments at the time of diagnosis is advisable; however, in this study, surgery was not delayed until after completion of the full 28-day course of doxycycline. The variability in scheduling of adjunctive therapy relative to anesthesia precluded assessment of its optimal timing, duration, or impact on perioperative risk.

When sterilization prior to adulticide therapy is not possible, anesthesia during the immediate post-adulticide period is not recommended. Anesthetic risk is likely higher during this period, because adulticide treatment unavoidably results in pulmonary thromboembolism and inflammation associated with worm death [13, 26, 29, 30]. Heartworms typically die within 1–4 weeks of melarsomine treatment. Thromboembolism is most common within the first 7–10 days, but can occur up to 4 weeks after worm death [7, 30]. Although a delay of 6 months between melarsomine administration and elective surgery has been suggested for dogs with more severe HWD, the optimal wait period after clinical recovery remains unknown [7]. In one shelter, HW-positive dogs with ASA classification scores greater than II were first treated for HWD and then surgically sterilized after a minimum 4-week wait period [15]. No specific guidance exists for dogs with less severe disease.

From an operational standpoint, these clinical findings carry important implications for shelter management. Beyond uncertainty regarding optimal anesthetic timing post-melarsomine, there are compelling operational reasons for shelters to sterilize dogs prior to melarsomine treatment. Delaying sterilization until after treatment would substantially increase length of stay and time in care. Scheduling spay–neuter procedures prior to melarsomine-based treatment also reduces the number of required veterinary visits compared with dogs adopted unaltered. These operational benefits must be weighed against potential risks in dogs with more advanced disease or comorbidities not represented in this cohort.

When placed in the context of published spay–neuter outcomes, the complication profile observed in this study is consistent with expectations, although it should be noted that these comparisons are indirect and should be interpreted cautiously due to differences in study design and populations. The vast majority of complications in this study were superficial surgical-site issues in male dogs that resolved without intervention or with short-term medical therapy. Complication rates were comparable to those reported for routine sterilization procedures in private and high-volume settings [31–36]. The all-cause perioperative mortality was 0%, consistent with low perioperative mortality in other high-volume settings, including 0.009% in another Florida shelter clinic and 0.08% in a Pennsylvania shelter clinic [35, 36]. The overall complication rate of 5% was similar to rates of 2.6–7% reported by others [34].

The study had several limitations, mostly associated with the retrospective nature of the medical record review, protocolized procedural medical notes in the electronic medical record, and limited postoperative follow-up for dogs that left the shelter following surgery. A control group of dogs without HWD was not included. However, the very low rate of potential HWD-related complications (0.3%) would yield a nonsignificant difference in a matched control group, even if no complications occurred in that group. As the purpose of this study was to evaluate safety of sterilization surgery in clinically stable dogs, these results should not be extrapolated to dogs with more serious HWD or other compromising conditions. Nor should these results be extrapolated to low-volume surgery settings, other anesthetic protocols, situations in which macrocyclic lactones and doxycycline are unavailable, or regions, where HWD is less prevalent. Although this is the largest study of surgery in dogs with HWD to date, statistical inference is limited by the retrospective, single‑site design.

## Conclusions

In summary, this retrospective study of 1,011 dogs demonstrated that surgical sterilization of dogs with Class 1 and 2 HWD was associated with low perioperative complication rates in this selected population, supporting its use in similar clinical settings prior to adulticide treatment. High-volume surgical techniques and unmodified standardized anesthetic protocols appear safe and acceptable in this population of dogs.

## Data Availability

Data supporting the main conclusions of this study are included in the manuscript.

## Abbreviations

Ag: Antigen
HQHVSN: High-quality high-volume spay–neuter
HW: Heartworm
HWD: Heartworm disease
IM: Intramuscularly
IV: Intravenously
Mf: Microfilariae
PO: Orally
